# A Single-Cell Transcriptome Profiling of Anterior Kidney Leukocytes From Nile Tilapia (*Oreochromis niloticus*)

**DOI:** 10.3389/fimmu.2021.783196

**Published:** 2021-12-28

**Authors:** Liting Wu, Along Gao, Lan Li, Jianlin Chen, Jun Li, Jianmin Ye

**Affiliations:** ^1^ Guangdong Provincial Key Laboratory for Healthy and Safe Aquaculture, Institute of Modern Aquaculture Science and Engineering, School of Life Sciences, South China Normal University, Guangzhou, China; ^2^ Guangdong Province Key Laboratory of Waterfowl Healthy Breeding, Zhongkai University of Agriculture and Engineering, Guangzhou, China; ^3^ School of Science and Medicine, Lake Superior State University, Sault Ste. Marie, MI, United States

**Keywords:** Nile tilapia, anterior kidney, leukocytes, single-cell transcriptome, cell markers

## Abstract

Teleost fish anterior kidney (AK) is an important hematopoietic organ with multifarious immune cells, which have immune functions comparable to mammalian bone marrow. Myeloid and lymphoid cells locate in the AK, but the lack of useful specific gene markers and antibody-based reagents for the cell subsets makes the identification of the different cell types difficult. Single-cell transcriptome sequencing enables single-cell capture and individual library construction, making the study on the immune cell heterogeneity of teleost fish AK possible. In this study, we examined the transcriptional patterns of 11,388 AK leukocytes using 10× Genomics single-cell RNA sequencing (scRNA-seq). A total of 22 clusters corresponding to five distinct immune cell subsets were identified, which included B cells, T cells, granulocytes, macrophages, and dendritic cells (DCs). However, the subsets of myeloid cells (granulocytes, macrophages, and DCs) were not identified in more detail according to the known specific markers, even though significant differences existed among the clusters. Thereafter, we highlighted the B-cell subsets and identified them as pro/pre B cells, immature/mature B cells, activated B/plasmablasts, or plasma cells based on the different expressions of the transcription factors (TFs) and cytokines. Clustering of the differentially modulated genes by pseudo-temporal trajectory analysis of the B-cell subsets showed the distinct kinetics of the responses of TFs to cell conversion. Moreover, we classified the T cells and discovered that CD3^+^CD4^−^CD8^−^, CD3^+^CD4^+^CD8^+^, CD4^+^CD8^−^, and CD4^−^CD8^+^ T cells existed in AK, but neither CD4^+^CD8^−^ nor CD4^−^CD8^+^ T cells can be further classified into subsets based on the known TFs and cytokines. Pseudotemporal analysis demonstrated that CD4^+^CD8^−^ and CD4^−^CD8^+^ T cells belonged to different states with various TFs that might control their differentiation. The data obtained above provide a valuable and detailed resource for uncovering the leukocyte subsets in Nile tilapia AK, as well as more potential markers for identifying the myeloid and lymphoid cell types.

## Introduction

Similar to mammals, teleost fish possess innate and adaptive immunity to protect themselves from microbial pathogen infection (1). It is the anterior kidney (AK) that serves as the main lymphoid organ for hematopoiesis in teleost fish instead of the bone marrow in mammals, with abundant cell populations divided into the myeloid lineage and the lymphoid lineage (2). The myeloid lineage mainly includes granulocytes, monocytes and macrophages, and dendritic cells (DCs), while the lymphoid lineage refers to B cells, T cells, and nonspecific cytotoxic cells (NCCs) (3–5). Based on the specific expressed cytokines and transcription factors (TFs), these cell populations can be classified into subsets with different functions (6–10).

There are four types of granulocytes (neutrophils, eosinophils, basophils, and mast cells) that have been identified with the basic criteria, such as ontogenetic, morphological, and functional (11). In zebrafish, studies on neutrophil response have been reported for several transgenic reporter lines, and this granulocyte subset has been marked with myeloperoxidase (MPX), the ortholog of mammalian myeloperoxidase (MPO) (12, 13). Neutrophils constitute the first line of defense against invading pathogens, migrating to sites of inflammation during normal immune responses to tissue injury and infection. Basophils are the least abundant leukocytes in most vertebrate species, but there is a natural abundance in teleost fish circulating peripheral blood with two distinct types of basophilic granules (14, 15). The CCAAT enhancer binding protein alpha (*CEBPA*) is the marker gene used for identifying fish basophils (15). Additionally, studies on adult zebrafish hematopoietic tissues discovered that all the highly expressed GATA binding protein 2 (*GATA2*) cells were eosinophils (6). Carboxypeptidase A5 (CAP5) was identified as a specific marker for zebrafish mast cells (16). Monocytes, macrophages, and DCs are components of the mononuclear phagocyte system (MPS) that clears invasive microbial pathogens during immune challenge and removes the apoptotic cell corpses through phagocytosis (17). A few pieces of information are available for fish monocytes alone, but most studies have been conducted on and represented as monocytes/macrophages because the monocytes in AK could quickly differentiate into macrophages once they interact with antigens or with the help of cytokines (18). Specific lysosomal markers for fish monocytes are not available, but markers used to identify macrophages are available, including macrophage expressed gene 1 (*MPEG1*), macrophage receptor with collagenous structure (Marco), macrophage mannose receptor 1 (CD206), CD68, and complement factor properdin (CFP) (19). Macrophages are divided into M1 and M2 populations based on the different expression patterns of TFs and cytokines and metabolism. The M1 phenotype includes signal transducers and activators of transcription 1 (STAT1), interleukin 12 (IL-12), and tumor necrosis factor alpha (TNF-α), whereas M2 macrophages typically produce arginase, legumain, and IL-10 (20–22). Teleost fish DCs have been identified in a few fish species (including rainbow trout and zebrafish) as antigen-presenting cells (APCs) with similar expression molecular markers to mammalian DCs, such as major histocompatibility complex (MHC) II, CD80/86, and CD83 (7, 19, 23). In Barramundi, it has been found that the DC-SCRIPT gene is a specific molecular marker for fish DCs, a homolog of the human *ZNF366* gene and the pufferfish *ZNF1* gene (24). In rainbow trout, the TFs *ZBTB46* and DC-SCRIPT/*ZNF366* have been identified to have potential involvement in DC maturation and activation (25).

Several research works have indicated that there are functional B-cell subpopulations that reside in AK, including pro/pre B cells, immature/mature B cells [(im)mat. B], activated B cells (act. B), and antibody-secreting cells [ASCs, including plasmablasts (PBs) and plasma cells (PCs)], which are regulated by the combinatorial expressions of B-cell-specific TFs such as E2A, IKAROS family zinc finger 1 (*IKZF1*), early B-cell factor-1 (*EBF1*), recombination activating 1 (Rag1), Rag2, paired box 5 (Pax5), B-lymphocyte maturation protein-1 (Blimp-1), and X-box binding protein 1 (XBP1) (9, 10, 26, 27). In zebrafish, it is indicated that the patterns of B-cell gene expressions are similar to those observed during mammalian B-cell differentiation in the major immunoglobulin M (IgM)-expressing B-cell subsets, such as pro B cells (Pax5^+^Rag2^+^IgM^−^), pre B cells (Pax5^+^Rag2^+^IgM^+^), (im)mat. B (Pax5^+^Rag2^−/lo^IgM^+^), and PCs (Pax5^−^Blimp-1^+^XBP1^+^CD40^+^) (10). Besides, fish T cells (CD3^+^) are identified as distinct T-cell (Tc) functional subsets according to the expression of either CD4 or CD8. T helper (Th) cells expressing CD4 (CD4^+^) are in the position of coordinating the immune system through the production of cytokines after activation and differentiation; they are divided into several subtypes, namely, Th1, Th2, Th6, Th9, Th17, and regulatory T cells (Tregs) (8, 28, 29). Th1 cells express three lineage-specific TFs: *STAT1*, *STAT4*, and *T-bet*. Th2 cells express two conserved TFs: *STAT6* and *GATA3*. Recently, IL-6 has been assigned as a signature cytokine of Th6 (30). Th17 cells are defined by the expressions of IL-17 and the TFs nuclear retinoic acid receptor (RAR)-related orphan receptor gamma (RORγ), *STAT1*, and *STAT3* (8). The Th22 subset was discovered to secrete IL-22, but not IL-17 (31). In pufferfish, a Treg-like subset with the phenotype CD4-2^+^CD25-like^+^Foxp3-like^+^ has been identified (32). Cytotoxic T cells (cytotoxic T lymphocytes, CTLs) expressing CD8 (CD8^+^) release cytotoxic factors that directly kill infected or abnormal cells and are involved in specific cell-mediated cytotoxicity in fish (33, 34). The heterogeneous differentiation and function of CD8^+^ T cells (Tc subsets) have been well established in mammals (35), as well as natural killer (NK) cells with similar functions to Tc that use perforin and granzyme to kill target cells (36, 37). NCCs are unique to fish and are considered to be functionally similar to mammalian NK cells, which have the ability to kill the target and affected cells through lysis (38–40), and NCC receptor protein 1 (*NCCRP1*) has been identified as the specific marker for this cell type (41).

Leukocytes are involved in innate and adaptive immunity, providing a critical first line of defense soon after pathogen infiltration, and in long-term immunological memory, providing whole of life protection (42). Although the specific markers described above have been used to identify fish cell subsets, limited knowledge about these cell types and the antibody reagents in teleost fish hampers a more in-depth characterization and separation of these cells. Several similarities exist between the immune systems of teleost fish and mammals, but there still remain unknown differences; more studies need to be performed on teleost fish. For example, a study on *MPEG1*, a specific marker for macrophages, has demonstrated, using single-cell RNA sequencing analysis (scRNA-seq), that it is expressed in a subpopulation of B cells in adult zebrafish (43). Moreover, in Nile tilapia, 10× Genomics scRNA-seq surprisingly revealed that *NCCRP1* is expressed not only in NCCs but also in other cell types (44). ScRNA-seq provides more detailed information of the different leukocyte subsets and updates our current knowledge about the immune cells in teleost fish. In this study, we analyzed the AK leukocytes with 10× Genomics scRNA-seq in order to identify leukocyte cell clusters. We aimed to provide more valuable information to elucidate the cell types and their potential functions. The identified cell markers for each subpopulation will also benefit the design of future novel specific marker antibodies that can be used to identify different cell types more accurately.

## Materials and Methods

### Experimental Fish

Nile tilapia (*Oreochromis niloticus*), with a mean weight of 250 g, were supplied by Guangdong Tilapia Breeding Farm (Guangzhou, China). Fish were maintained in 300-L fiber glass-reinforced plastic tanks at the Aquaculture Breeding Center of the South China Normal University, with an automatic filtering aquaculture system at 28 ± 2°C and a 12:12-h light/dark photoperiod. The fish were fed a commercial diet once a day. No clinical signs were observed during feeding. All animal protocols were reviewed and approved by the University Animal Care and Use Committee of the South China Normal University.

### Isolation of Tilapia Anterior Kidney Leukocytes

**Figure 1:**
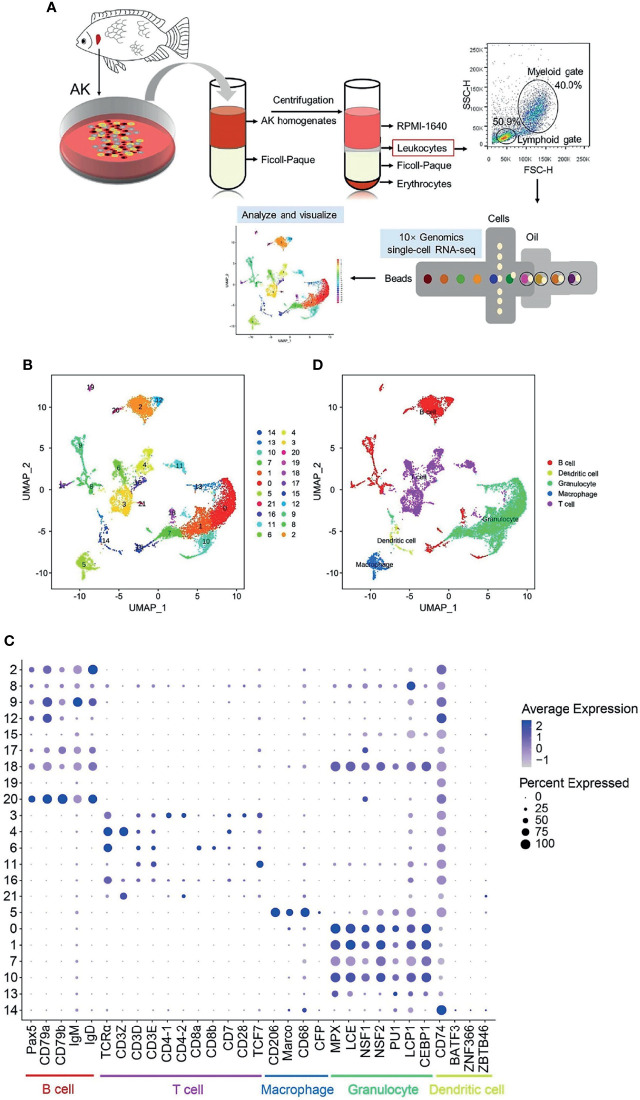
Single-cell RNA sequencing (scRNA-seq) analysis of Nile tilapia anterior kidney (AK) leukocytes. **(A)** Experimental workflow for the scRNA-seq of leukocytes from Nile tilapia AK, including cell separation, flow cytometry detection, and 10× Genomics Chromium 3′ scRNA-seq and data visualization. **(B)** A total of 22 cell clusters were identified and shown with a uniform manifold approximation and projection (UMAP) space. **(C)** Bubble chart of the putative marker genes for B cells, T cells, macrophages, granulocytes, and dendritic cells (DCs) in the cell clusters. The *dot size* and *color intensity* represent the gene expression percentage and the average expression levels of the cells within a cluster, respectively. **(D)** Identification of the putative cell types based on the expressions of marker gene in both mammals and fish.

Nile tilapia were anesthetized with 3-aminobenzoic acid ethyl ester (MS-222; Aladdin, Shanghai, China), and blood was extracted from the caudal vein with a heparinized syringe to prevent blood pollution to the AK. Leukocytes from the AK were obtained as previously described, with some modifications (45). Briefly, the AK were dissected with aseptic dissection tools and placed into a sterile plastic culture dish containing 5 ml RPMI-1640 (Gibco, Grand Island, NY, USA) with 100 U/ml penicillin G and 100 μg/ml streptomycin (Sigma, St. Louis, MO, USA). Tissues were gently aspirated using a 1-ml syringe until no chunks of tissues existed. Then, the single-cell suspension was filtered with a 70-μm filter (NEST, Wuxi, China) to remove the tissue fragments and transferred into a sterile tube before RPMI-1640 (Gibco, Grand Island, NY, USA) was added to total volume 10 ml. The suspension was gradually layered upon the same volume of Ficoll-Paque^®^ PREMIUM (density = 1.077 g/ml) (GE Healthcare, Chicago, IL, USA) in 50-ml centrifuge tubes and then centrifuged at 500 × *g* for 40 min at 4°C. Leukocytes were collected from the interface layer and washed three times with the RPMI-1640 medium. Cell quantity and viability were determined using 0.4% trypan blue (Sigma, St. Louis, MO, USA), which showed that more than 98% were living cells. Subsequently, the cells were resuspended to a concentration of 1 × 10^6^ cells/ml in RPMI-1640. The mixed leukocytes were prepared from three fish AKs and used for sequencing. Meanwhile, the cells were detected by flow cytometry (FCM) on BD FACSAria III (BD Biosciences, Franklin Lakes, NJ, USA) equipped with DIVA software to ensure detection of the main lineages (**Figure 1A**).

### Library Construction and Sequencing

The isolated AK leukocytes, 1,000 cells/µl, were used for scRNA-seq on a 10× Genomics Chromium instrument (10× Genomics, Pleasanton, CA, USA). Firstly, a total of ~20,000 cells were loaded into the chips of the Chromium™ Single Cell 3′ Gel Bead Kit (10× Genomics, Pleasanton, CA, USA) and subjected to the Chromium instrument to generate single-cell Gel Bead-In Emulsions (GEMs) following the manufacturer’s instructions. The GEMs were then subjected to library construction using the Chromium™ Single Cell 3′ Library Kit (10× Genomics, Pleasanton, CA, USA). Briefly, upon dissolution of the Single Cell 3′ Gel Bead in a GEM, primers containing an Illumina^®^ Read 1 sequencing primer, a 16-nt 10× barcode, a 10-nt unique molecular identifier (UMI), and a poly-dT primer sequence were released and mixed with the cell lysate and master mix. Incubation of the GEMs then produced barcoded full-length complementary DNA (cDNA) from poly-adenylated messenger RNA (mRNA). After incubation, the GEMs were broken and the pooled fractions were recovered. Silane magnetic beads were used to remove leftover biochemical reagents and primers from the post-GEM reaction mixture. The barcoded full-length cDNA was then amplified by PCR (Bio-Rad, Hercules, CA, USA) to generate sufficient mass for library construction. The Single Cell 3′ Protocol produces Illumina-ready sequencing libraries. The library synthesis and RNA-seq were completed with Illumina HiSeq 4000 by the Gene Denovo Biotechnology Co., Ltd. (Guangzhou, China).

### Initial Data Quality Control, Cell Filtering, and Data Normalization

The Cell Ranger Single Cell Software Suite (version 6.1; http://software.10xgenomics.com/single-cell/overview/welcome) (10× Genomics, Pleasanton, CA, USA) was used to perform quality control, barcode processing, and Single Cell 3′ gene counting. For removal of low-quality sequences with barcodes and UMIs, the raw data were first demultiplexed into the FASTQ format with the bcl2fastq software and then aligned in the Nucleotide Sequence Database (https://www.ncbi.nlm.nih.gov/genbank/) with the NCBI Basic Local Alignment Search Tool (BLAST). A total of 394,170,826 clean reads were obtained based on the transcriptome of 11,743 cells, achieving an average read of 33,566 per cell. The Q30 bases in barcode, RNA read, and UMIs were 95.6%, 90.1%, and 95.2%, respectively.

The FASTQ files were mapped to the Nile tilapia reference genome (ASM185804v2) using the STAR RNA-Seq aligner. Once aligned, barcodes associated with these reads–UMIs were subjected to filtering and correction. For UMI tag counting, the 10× Genomics pipeline Cell Ranger was used to generate single-cell gene counts for this library. Only the confidently mapped, non-PCR duplicates with valid barcodes and UMIs were used to generate the gene–barcode matrix. The Seurat R package (version 4.0.4) (46, 47) was used for quantity control and filtering of abnormal cells according to their molecular counts. The criteria used to filter cells were as follows: 1) less than 200 but more than 4,000 gene counts per cell; 2) UMI counts >30,000 per cell; and 3) percentage of mitochondrial (Mito) genes of >10%. Data normalization was performed with a global-scaling normalization method, “LogNormalize,” after removal of unwanted cells from the dataset. This normalizes the gene expression measurements for each cell with the following formula: Gene A expression level = log(1 + (UMI A ÷ UMI Total) × 10,000).

### Transcriptome Dimensionality Reduction, Cell Clustering, Visualization, and Marker Gene Identification

The filtered and normalized dataset was examined using canonical correlation analysis and then the data were integrated. The integrated data were normalized by the *Z*-score and then subjected to dimensionality reduction using principal component analysis (PCA) to reduce the variables. Subsequently, a graph-based clustering approach was used to cluster cells. This setup was also applied to define the nearest neighbors among cells with the *k*-nearest neighbor (KNN) method using the findNeighbors function. To group the cells into different subsets according to their expression levels, the Find Cluster tool was applied using the Louvain algorithm with the resolution set as 0.5, allowing the correct definition of clearly separated clusters. Based on the results of the cell subgroup classification, the single-cell subgroup classification results were further visualized by uniform manifold approximation and projection (UMAP) with the Loupe Cell Browser software and Seurat R package (version 4.0.4). Data clustering was performed using the Seurat R package. The identification of genes showing differential expressions associated with a specific cluster was performed with the known specific cell gene expressions.

### Single-Cell Pseudo-Time Analysis

Further exploration of the single-cell trajectories in B and T cells was conducted with a matrix of cells and gene expressions using Monocle 3 (48). Monocle reduced the space down to one with two dimensions and ordered the cells. We identified the key genes related to the development and differentiation process and grouped those with similar trends in expression, reasoning that such groups may share common biological functions and regulators. Differential gene testing for the pseudo-time analysis was based on the previously identified cell clusters. The cells were ordered and visualized in the trajectory in the reduced dimensional space, in a tree-like structure (including tips and branches).

### GO and KEGG Enrichment Analyses

For the analysis of the differentially expressed genes (DEGs), Gene Ontology (GO) enrichment analysis was performed on the DEGs by comparing with the genome background and filtering the DEGs that correspond to biological functions. All peak-related genes were mapped to GO terms in the GO database (http://www.geneontology.org/), gene numbers were calculated for every term, and the significantly enriched GO terms in DEGs compared to the genome background were defined using a hypergeometric test. The calculated *p*-values were false discovery rate (FDR) corrected, and FDR ≤ 0.05 was taken as a threshold to define the significantly enriched GO terms in DEGs.

Genes interact with each other and play certain biological functions. Pathway-based analysis helps in further understanding the biological functions of a gene. Kyoto Encyclopedia of Genes and Genomes (KEGG) (https://www.kegg.jp/) pathway enrichment analysis identified the significantly enriched metabolic pathways or signal transduction pathways in DEGs by comparing with the whole genome background. The calculated *p*-value was FDR corrected and an FDR ≤ 0.05 was used as the threshold. Pathways meeting this criterion were defined as significantly enriched pathways in DEGs.

## Results

### Overall Characteristics of the Cell Cluster Composition of Tilapia AK Leukocytes

AK leukocytes were obtained from healthy Nile tilapia using Ficol-Paque^®^ PREMIUM density gradient media (density, 1.077 g/ml) (GE Healthcare, Chicago, IL, USA), and cell viability was checked and confirmed to be about 98% with microscopic examination. Single-cell cDNA libraries were sequenced using the two-terminal sequencing mode of the Illumina HiSeq 4000 sequencing platform. After quality control and mapping using the Cell Ranger software, a total number of 394,170,826 reads in 11,743 single cells were acquired, with mean reads per cell of 33,566 and median genes per cell of 1,024.

The scRNA-seq data were then analyzed to identify the effective cell number (**Supplementary Figure S1A**) and determine the total number of genes (nFeature_RNA), the total number of UMIs (nCount_RNA), and the percentage of reads mapping the Mito genes (Percent.mito) (**Supplementary Figure S1B**). Abnormal cells were filtered when 1) the gene counts were less than 200 but more than 4,000 per cell, 2) the UMI counts were >30,000 per cell, and 3) the percentage of Mito genes was >10% (after filtering; shown in **Supplementary Figure S1B**). The relationship between nCount_RNA and nFeature_RNA and that between nCount_RNA and percent_mito before and after filtering (**Supplementary Figure S1C**) were shown as well. After filtering, a total of 11,388 cells were retained for subsequent analysis.

The overall experimental workflow for scRNA-seq of the leukocytes from Nile tilapia AK is shown in **Figure 1A**. A total of 22 cell clusters (clusters 0–21) were characterized (**Figure 1B**). Based on the known cell markers (**Supplementary Table S1**), 3,176 cells (accounting for 27.9% of the total) from clusters 2, 8, 9, 12, 15, 17, 18, 19, and 20 were categorized into B cells, and 2,857 cells (accounting for 25.1% of the total) from clusters 3, 4, 6, 11, 16, and 21 were classified into T-cell populations. Cells in clusters 14 and 5 were classified as DCs (208 cells, 1.8% of the total cells) and macrophages (564 cells, 5.0% of the total cells), respectively. The remaining 4,583 cells, included in clusters 0, 1, 7, 10, and 13, were classified as granulocytes, with a relative percentage of 40.2% (**Table 1** and **Figure 1C**). Clustering of the identified cell types was annotated with UMAP and shown in **Figure 1D**.

**Table 1:** Identified cell types and the corresponding cell numbers (percentages) in Nile tilapia anterior kidney leukocytes.

Cell type	No. of cells (%)
B cells	3,176 (27.9)
T cells	2,857 (25.1)
Macrophages	564 (5)
Granulocytes	4,583 (40.2)
Dendritic cells	208 (1.8)
Total	11,388 (100)

### Granulocyte Subset Analysis in the Identified Granulocytes

It is known that granulocytes contain neutrophils, eosinophils, basophils, and mast cells (11). Therefore, the identified granulocytes were further classified into eight clusters with a resolution of 0.5 (**Supplementary Figure S2A**). The top 5 DEGs for each cluster are shown in a heatmap (**Supplementary Table S3** and **Figure S2B**), and the reported marker genes, including *EPX*, *CEBPA*, and *GATA2* (*GATA2a* and *GATA2b*), were used to identify neutrophils, basophils, and eosinophils, respectively (**Supplementary Table S2** and **Figure S2C**). Moreover, *NCCRP1*, neutrophil cytosolic factor 1 (NSF1), NSF2, high choriolytic enzyme 1 (HCE1), low choriolytic enzyme (LCE), BCL2 family member b (MCL1b), CD66, colony stimulating factor 1 receptor (*CSF1R*), macrophage-expressed gene 1 (*MPEG1*), Spi-1 proto-oncogene b (SPI1b/PU1), *GATA2a*, *GATA2b*, histidine decarboxylase (HDC), and Fc receptor gamma subunit (*FCER1G*) were used to identify the granulocyte subsets further (**Supplementary Table S2** and **Figure S2D**). However, the expression patterns among these clusters were similar, resulting in not being able to distinguish the different granulocytes based on these genes.

### Macrophage Subset Analysis in the Identified Macrophages

The macrophage lineage cells present a series of functional specializations in vertebrates, and different subgroups (M1 and M2) have been identified and studied in teleost fish as in mammals (49, 50). The identified macrophages were further classified into four clusters with a resolution of 0.5 (**Supplementary Figure S3A**). The top 5 DEGs for each cluster are shown in a heatmap (**Supplementary Table S4** and **Figure S3B**), and the reported cell subset gene markers, including *STAT1*, *TNFα*, *IL-12*, *Arginase-1*, *Arginase-2*, legumain, and *IL-10*, were used to identify M1 and M2 cells (**Supplementary Table S2** and **Figure S3C**). Moreover, related genes, such as microphthalmia-associated transcription factor (*MITF*), transcription factor 3b (*TCF3b*), *CEBPA*, transcription regulator protein (*BACH1*), cellular repressor of E1A stimulated gene 1 (*CREG1*), *GATA6*, Spi-C transcription factor (*SPIC*), *CCL20b*, complement factor B (*CFB*), microfibril-associated glycoprotein 4 (*MFAP4*), interleukin-12 subunit beta (*IL-12β*, *IL-12p40*), interleukin-12 subunit alpha (*IL-12p35*), *IL-34*, *CD209*, *CSF1R*, and *CSF2Rb*, were used to identify macrophage populations (**Supplementary Table S2** and **Figure S3D**). However, most of these marker genes were low expressed and those with high expression were similar in the four clusters. It seemed, therefore, that M1 and M2 macrophages cannot be identified in these clusters.

### Dendritic Cell Subset Analysis in the Identified Dendritic Cells

DCs have been considered as the most powerful professional APCs in mammalian species (51). Different from mammalian DCs, teleost fish DCs have only been identified in a few fish species (including rainbow trout and zebrafish) and without any detailed information for further subtype characterization (7, 19, 23). It is valuable to identify the teleost fish DC subsets to determine whether they can be classified with the known gene markers in mammals. Therefore, we further classified the DCs into three clusters with a resolution of 0.5 (**Supplementary Figure S4A**). The top 5 DEGs for each cluster are shown in a heatmap (**Supplementary Table S5** and **Figure S4B**), and the reported cell subset gene markers were used to identify the DC subsets (**Supplementary Table S2** and **Figure S4C**). Although the gene markers in the three subsets did not show the anticipated results, the antigen-presenting molecules were highly expressed in cluster 1. We further analyzed the top 3 most significant GO terms within the biological process (BP) category for cluster 1 and found that the immune system process, regulation of metabolic process, and intracellular signal transduction were enriched (**Supplementary Table S6**).

### Identification of B-Cell Subsets and Analysis of Single-Cell Trajectories

**Figure 2:**
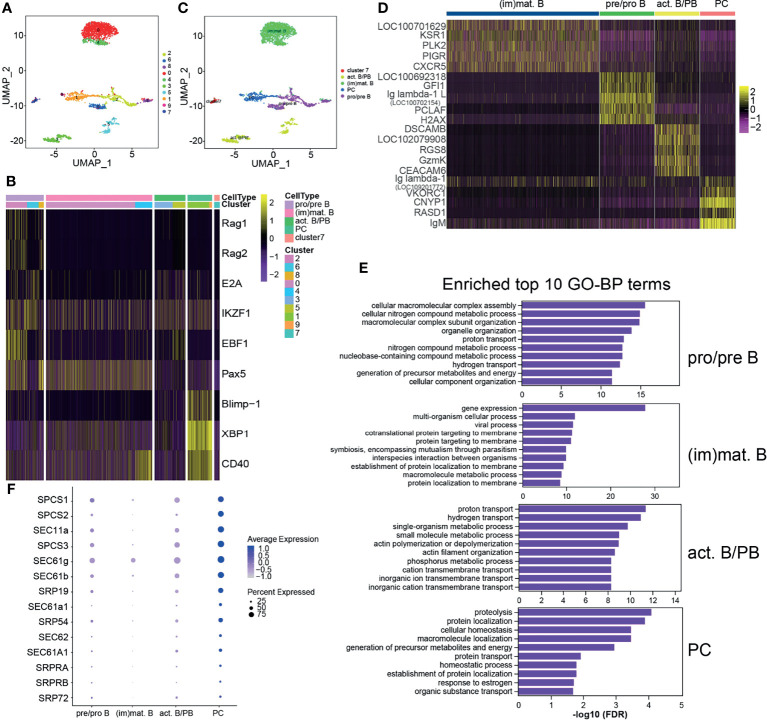
Heterogeneous subsets in B cells. **(A)** The identified whole B cells were classified into 10 different clusters and shown with a uniform manifold approximation and projection (UMP) space. The clusters were obtained with a resolution of 0.3. **(B)** Heatmap of the different expressions of the genes used to identify the B-cell subsets. The clusters were identified into four B-cell subsets: pre/pro B, immature/mature B cells [(im)mat. B)], activated B cell and plasmablasts (act. B/PBs), and plasma cells (PCs). The remaining small group in cluster 7 was not identified. **(C)** Distribution of the identified B-cell subsets in UMAP space. **(D)** Heatmap of the top 5 most significant specific genes for each B-cell subset. **(E)** The top 10 most significant Gene Ontology (GO) terms within the biological process (BP) category for each B-cell subset. **(F)** Bubble chart of the enriched protein transport genes in B-cell subsets.

Teleost fish B cells contain distinct B-cell subsets during teleost immune development, similarly to mammals, and TFs play roles in vertebrate B-cell development (9, 10). The identified B cells were further classified into 10 clusters with a resolution of 0.3 (**Figure 2A**). Different B-cell-specific expression markers were used to identify the B-cell subsets, and it was demonstrated that clusters 2, 6, and 8 were pro/pre B, clusters 0 and 4 were (im)mat. B, clusters 3 and 5 were act. B/PBs, and clusters 1 and 9 were PCs; cluster 7 did not classify with any of the categories (**Supplementary Table S7** and **Figures 2B, C** **)**. The top 5 most significant subset-specific genes for each B-cell subset are shown in a heatmap (**Supplementary Table S8** and **Figure 2D**). It is noteworthy that *CXCR5* and *IgM* were the most significant marker transcripts in (im)mat. B and PCs, respectively. We analyzed the top 10 most significant GO terms within the BP category for each B-cell subset and discovered that metabolic process was enriched in every B-cell subset, demonstrating that metabolism is related to B-cell development (**Supplementary Table S9** and **Figure 2E**). It is particularly important for humoral immune response that plasma cells synthesize and secrete tremendous amounts of antibodies (26, 52). In PCs, one of the most significant BP categories and the most significant KEGG pathway (**Supplementary Table S10** and **Figure S5A**) was enriched in protein transport (**Supplementary Table S11**), as shown in the heatmap (**Figure 2F**), implying that these genes may regulate antibody secretion in PCs. Moreover, antigen processing and presentation was significantly enriched in (im)mat. B (**Supplementary Table S12** and **Figures S5A, B**). It is widely recognized that teleost fish B cells have antigen-presenting ability (53), and these data implied that (im)mat. B cells may exert their main function among the B-cell subsets.

**Figure 3:**
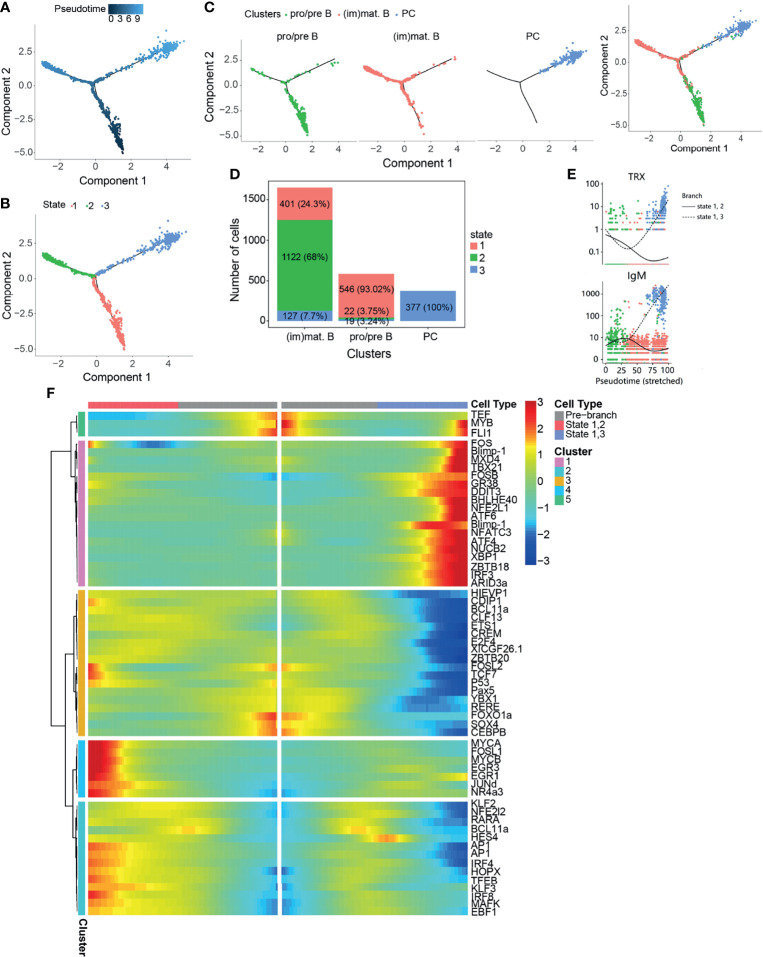
Pseudo-temporal analysis of B-cell development using Monocle. **(A)** Trajectories of pre/pro B, immature/mature B cells [(im)mat. B], and plasma cells (PCs) applying pseudo-time analysis. **(B)** Distribution of the three trajectory states. **(C)** Distribution of the three B-cell subsets in the trajectory. **(D)** Cell numbers and percentages of each B-cell subset in the trajectory branches. **(E)** Monocle analysis revealing the progressive expressions of thioredoxin (TRX) and IgM across pseudo-time in two branches. **(F)** Heatmap of the branch-dependent transcription factors (TFs) in the B-cell trajectory branch.

In order to reveal the potential relationships among the B-cell subsets during B-cell development, pseudo-temporal analysis was performed in B-cell subsets, including pro/pre B, (im)mat. B, and PCs (**Figure 3**), using Monocle. Distinct pseudo-time trajectory states in B-cell subsets were defined and are shown in **Figures 3A–C**. Among them, pro/pre B cells, being the starting point of differentiation, was in state 1, followed by (im)mat. B (state 2) and PCs (state 3). The identified PCs were in a single branch, and pro/pre B were shown to gradually differentiate into (im)mat. B (**Figure 3D**). According to the analysis of the top 10 genes branching differential genes, thioredoxin (TRX) and IgM were found to be highly expressed in state 3 cells (PCs) (**Figure 3E**). To further explore the regulation of B-cell differentiation, we analyzed the differently expressed TFs (**Supplementary Figure S5** and **Figure 3F**). Pseudo-temporal analysis revealed a trajectory of gene expression associated with B-cell differentiation in cells in states 1, 2, and 3 (**Supplementary Table S13** and **Figure 3F**), which provided us the potential key TFs that regulate teleost B-cell development.

### Identification of T-Cell Subsets and Analysis of Single-Cell Trajectories

**Figure 4:**
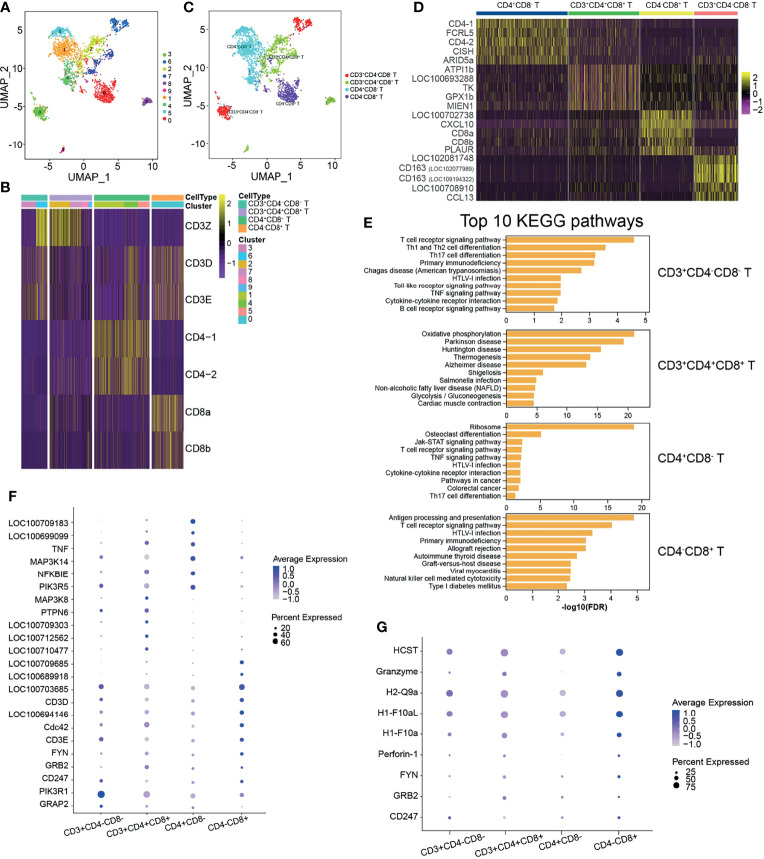
Heterogeneous subsets in T cells. **(A)** The identified whole T cells were classified into 10 clusters and shown with a uniform manifold approximation and projection (UMP) space. The clusters were obtained with a resolution of 0.5. **(B)** Heatmap of the different expressions of the genes used to identify the T-cell subsets. T cells were identified into four T-cell subsets: CD3^+^CD4^−^CD8^−^, CD3^+^CD4^+^CD8^+^, CD4^+^CD8^−^, and CD4^−^CD8^+^. **(C)** Distribution of the identified T-cell subsets in UMAP space. **(D)** Heatmap of the top 5 most significantly expressed genes for each T-cell subset. **(E)** The top 10 most significant Kyoto Encyclopedia of Genes and Genomes (KEGG) pathways for each T-cell subset. **(F)** Bubble chart of the related genes in the enriched pathway of T-cell receptor signaling in T-cell subsets. **(G)** Bubble chart of the related genes in the enriched pathway of natural killer cell-mediated cytotoxicity in T-cell subsets.

In mammals, T cells are divided into several subsets and play distinct functions in both humoral and cell-mediated immune responses. Teleost fish T cells developing similar functions to those known in mammals have been reported (8, 28, 29, 33). Based on the reported specific genes related to various T-cell subsets, we attempted to identify the T-cell subsets further. The identified T cells were classified into 10 clusters with a resolution of 0.5 (**Figure 4A**). Different T-cell-specific expression markers were used to identify the T-cell subsets, which demonstrated that cells in clusters 3 and 6 were CD3^+^CD4^−^CD8^−^, those in clusters 2, 7, 8, and 9 were CD3^+^CD4^+^CD8^+^, cluster 1, 4, and 5 cells were CD4^+^CD8^−^, and those in cluster 0 were CD4^−^CD8^+^ T cells (**Figures 4B, C**). The top 5 most significant subset-specific genes for each T-cell subset are shown in a heatmap (**Supplementary Table S14** and **Figure 4D**). The CD4^+^CD8^−^ T-cell subset highly expressed CD4-1 and CD4-2, while CD8-1 and CD8-2 were highly expressed in the CD4^−^CD8^+^ T-cell subset (**Figure 4D**). To further explore differences, we analyzed the top 10 most significant GO terms within the BP category for each T-cell subset and discovered that macromolecule metabolic process was enriched in the CD4^+^CD8^−^ T-cell subset, while the regulation of cellular metabolic process was the enriched BP category in CD4^−^CD8^+^ T cells (**Supplementary Table S15** and **Figure S7**). KEGG pathways analysis provided more information on the T-cell subsets, which enriched a lot of pathways related to T-cell subset functions (**Supplementary Table S16** and **Figure 4E**). Although the T-cell receptor signaling pathway was enriched in the T-cell subsets, except for CD3^+^CD4^+^CD8^+^ T cells, the highly expressed genes in the different T-cell subsets differed from each other (**Supplementary Table S17** and **Figure 4F**). It is known that CTLs expressing CD8 (CD8^+^) release cytotoxic factors that kill the infected or abnormal cells by secreting perforin/granzyme and are involved in specific cell-mediated cytotoxicity in fish (33, 34). In the CD4^−^CD8^+^ T-cell subset, NK cell-mediated cytotoxicity was the enriched pathway, and granzyme and perforin-1 were enriched, as anticipated (**Supplementary Table S18** and **Figure 4G**).

It is known that the CD4^+^ Th subtypes, namely, Th1, Th2, Th6, Th9, Th17, Th22, and Tre cells, perform different immune functions subsequent to their differentiation from naive T cells (8, 29). For the CD8^+^ CTLs, the Tc subtypes Tc1, Tc2, Tc9, and Tc17 have diversity functions and differentiation programs (54). Four clusters were obtained from the identified CD4^+^CD8^−^ T cells, and we attempted to identify the Th subtypes based on the reported cytokines and TFs. However, it appeared that the CD4^+^CD8^−^ T-cell subset cannot be identified into Th subtypes due to the specific genes being expressed in all clusters and not concentrated in a certain cluster (**Supplementary Figure S8A**). Similar results were shown for CD4^−^CD8^+^ T cells, where two clusters were classified but none of Tc1, Tc2, Tc9, and Tc17 can be identified completely based on the specific cytokines and TFs (**Supplementary Figure S8B**).

**Figure 5:**
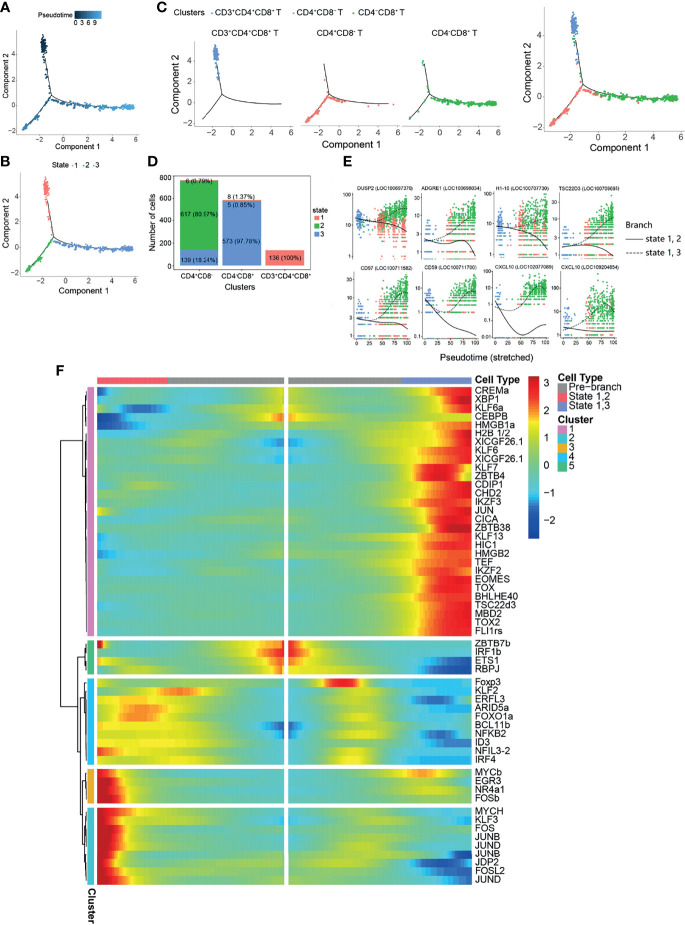
Pseudo-temporal analysis of T-cell development with Monocle. **(A)** Trajectories of CD3^+^CD4^+^CD8^+^, CD4^+^CD8^−^, and CD4^−^CD8^+^ with pseudo-time analysis. **(B)** Distribution of the three trajectory states. **(C)** Distribution of the three T-cell subsets in the trajectory. **(D)** Cell numbers and percentages of each T-cell subset in the trajectory branches. **(E)** Monocle analysis revealing the progressive expressions of dual specificity protein phosphatase 2 (*DUSP2*), adhesion G protein-coupled receptor E1 (*ADGRE1*), histone H1.10 (*H1-10*), TSC22 domain family protein 3 (*TSC22D3*), *CD97*, *CD59*, and C–X–C motif chemokine 10 (*CXCL10*) across pseudo-time in two branches. **(F)** Heatmap of the branch-dependent transcription factors (TFs) in the T-cell trajectory branch.

To explore the relationship between CD3^+^CD4^+^CD8^+^, CD4^+^CD8^−^, and CD4^−^CD8^+^ T cells, we performed a pseudo-temporal trajectory analysis in order to reveal a trajectory of gene expressions associated with the functional changes in these cells (**Figure 5**). Distinct T-cell subset pseudo-time trajectory states are defined and shown in **Figures 5A–C**. CD3^+^CD4^+^CD8^+^ T cells were defined as state 1, followed by CD4^+^CD8^−^ T cells (state 2) and CD4^−^CD8^+^ T cells (state 3) (**Figures 5B–D**). Analysis of the top 10 genes branching differential genes revealed that dual specificity protein phosphatase 2 (*DUSP2*), adhesion G protein-coupled receptor E1 (*ADGRE1*), histone H1.10 (*H1-10*), TSC22 domain family protein 3 (*TSC22D3*), *CD97*, *CD59*, and C–X–C motif chemokine 10 (*CXCL10*) were highly expressed in state 3 cells (CD4^−^CD8^+^ T cells), but their expressions decreased in state 2 cells (CD4^+^CD8^−^ T cells) (**Figure 3E**). To further explore the changes in differentiating T-cell subsets, the TFs were analyzed (**Supplementary Figure S9** and **Figure 5F**). The results showed different expression patterns of the TFs associated with CD4^+^CD8^−^ (states 1 and 2) and CD4^−^CD8^+^ (states 1 and 3) T cells (**Supplementary Table S19** and **Figure 5F**).

## Discussion

Various leucocytes are enriched in teleost fish AK executing immune functions comparable to those observed in mammals. The separation and enrichment of specific types of leukocytes are a crucial step in exploring their functions. However, limited knowledge on teleost fish immune cells, as well as the lack of specific antibody-based reagents and cell lines for non-model organisms, makes an accurate analysis of certain cell types difficult. In this study, the profiling of 11,388 cells from Nile tilapia provided transcriptional insights into how and what the leukocyte types consist of in healthy tilapia. We annotated five cell types (B cells, T cells, granulocytes, macrophages, and DCs) from 22 clusters and highlighted the B/T-cell subsets for cell developmental trajectories, which illustrated the potential temporal TFs that control B/T-cell differentiation.

The isolation of leukocytes from the AK has been widely explored by centrifugation in density gradients for a long time. Various mononuclear cell separation medium, mainly including Sepracell-MN (no longer manufactured), Percoll (GE Healthcare, Chicago, IL, USA), Histopaque^®^-1077 (Sigma, St. Louis, MO, USA), and Ficoll-Paque^®^ PREMIUM (GE Healthcare, Chicago, IL, USA), have been used to isolate the different cell types in teleost fish (26, 55, 56). Among these, continuous Percoll density gradients are used to isolate different types of immune cells (monocytes/macrophages, NCCs, and DCs) roughly (44, 57–59), but Histopaque^®^-1077 and Ficoll-Paque^®^ PREMIUM are used to obtain the leukocytes (a mixture of various immune cells, but not referring to certain cells) (26, 60). Leukocytes in *Oncorliynchus mykiss* AK tissues separated on Sepracell-MN have been demonstrated to contain 47% lymphocytes, 35% neutrophils, and 11% macrophages (55). The cell types identified in this study indicated that the obtained leukocytes from tilapia AK consisted of about 53% lymphoid cells and 47% myeloid cells (**Figure 1** and **Table 1**), which appeared to be consistent with the distribution in the FCM (**Figure 1A**) and the proportion distribution in trout.

In this study, myeloid cells were annotated using the expressions of marker genes and were classified into granulocytes, macrophage, and DCs (**Figure 1C**). Surprisingly, none of them can be identified into subsets based on the known gene markers (**Supplementary Figures S2**-**S4**). It is known that the differentiation or polarization (e.g., macrophages can be polarized into M1 or M2 macrophages) of these immune cells is trigged by the corresponding cytokines and TFs under stress, immune, and inflammatory responses (1). Therefore, the possible reason for the inability to identify these cell subtypes is that not every cell subtype exists in healthy Nile tilapia. The myeloid clusters obtained in this study will provide useful information for further studies on the inflammatory response of Nile tilapia. Until now, although it has been taken as a model to explore teleost fish immunity, studies on Nile tilapia cell types have been limited. The marker genes used to identify granulocytes, macrophages, and DCs were noteworthy for identifying these cells, but additional work is needed to validate the accuracy of the results. In granulocyte clusters, the top 5 most highly significant subset-specific genes in cluster 6 included *CD79* and *IgM* (B-cell maker genes) and those in cluster 7 included *CD3E* (T-cell marker gene) (**Supplementary Figure S2B**). It is unknown whether or not this phenomenon is a result of the doublets produced during isolation with chromium. This phenomenon cannot be explained using current knowledge about teleost fish immune cells; further efforts are required.

The lymphocytes identified in this study included B and T cells, but not NCCs (**Figure 2**). The reported tilapia scRNA-seq revealed different NCC subsets post-poly I:C intraperitoneal injection with 51%/34% Percoll density gradient (44). The proven result of NCCRP1 being expressed in the identified NCCs and macrophages was consistent with our study on granulocytes (**Supplementary Figure S2C**), suggesting that other specific markers were needed for NCC identification. The successfully identified B-cell subsets demonstrated that teleost AK comprised B-cell subsets with different differentiation stages that may exert different functions (**Figure 2**). Different metabolic processes were enriched in the B-cell subsets, suggesting that these subsets have active activities involved in immune processes in AK (**Figure 3E**). It is widely accepted that mature B cells have the ability to process and present antigens to Th cells (61). Antigen processing and presentation was the enriched pathway in (im)mat. B cells, implying that teleost (im)mat. B cells may play a critical role in B-cell antigen presentation. The related TFs in the B-cell subset developmental trajectories demonstrated that B-cell differentiation in teleost fish shows similarities and differences when compared to B-cell development in mammals (62, 63). It indicated that *RAG1*, *RAG2*, *E2A*, *IKZF1*, *EBF1*, *Pax5*, *Blimp-1*, and *XBP1* were the key TFs controlling tilapia B-cell differentiation, which is similar to mammalian B-cell differentiation (**Figures 3** and **4**). Recently, in rainbow trout blood B cells, different B-cell subsets in different stages of maturation or differentiation have been revealed (64). We discovered that the B-cell subsets we identified were consistent with the blood B-cell subsets to a certain extent, including the TFs and cytokines. The immunoglobulins in Nile tilapia include IgM (LOC106096470), IgD (LOC100709195), and IgT (LOC100701592). Among them, the expression of IgT was unexpectedly low in the B-cell subsets (for this reason, we have not included the corresponding data in **Figure 1C**). It is known that the degree of maturation of B cells in mammals is associated with the presence or absence of IgD; however, it was concluded that the membrane and secreted types of IgD and IgM were expressed differently in the B-cell subsets. Therefore, we have not added these into identifying B-cell subsets. However, it appears that the membrane or secreted types of IgD and IgT cannot be identified further in the Nile tilapia reference genome (ASM185804v2). As a result, it appears infeasible to identify the maturation degree of B cells here based on the expressions of IgD/IgM. It is especially interesting to explore and discuss the IgT subset maturation in fish, and such work can be carried out in the mucosal immunity system, such as the skin and gills, which are enriched in IgT^+^ cells. More information is worthy of further study and exploitation.

The thymus is a crucial organ for the development of T lymphocytes from early thymocyte progenitors to functionally competent T cells (28). Surprisingly, the data presented here indicated that CD3^+^CD4^−^CD8^−^ T cells exist in teleost fish AK, and *CD163* (a monocyte/macrophage-specific membrane marker) was the most significant subset-specific gene (**Figure 4D**). Existing knowledge on teleost fish T cells cannot be used to explain this phenomenon and should be further explored. CD3^+^CD4^+^CD8^+^ T cells will develop and mature into CD4^+^CD8^−^ or CD4^−^CD8^+^ T cells. At the onset of positive selection, CD8 transcriptional expression is shut down in thymocytes, which is independent of whether they received MHC class I or class II signals. This results in the appearance of intermediate CD4^+^CD8^−^ cells expressing an MHC II-restricted T-cell receptor (TCR). When CD4^+^CD8^−^ cells express an MHC I-restricted TCR, TCR signaling is interrupted, leading to the downregulation of CD4 and the specification of CD4^−^CD8^+^ cytotoxic T cells (65). Naive T cells are activated following antigen or pathogen exposure and then differentiate into functional T-cell subsets (8, 28, 33, 34). This might the reason for the phenomenon that neither CD4^+^CD8^−^ nor CD4^−^CD8^+^ T cells can be classified into the Th or Tc subsets further in healthy tilapia (**Supplementary Figure S8**). Studies on the differentiation of T cells are limited, and the pseudo-temporal analysis revealed that the key TFs might control the development of the T-cell subsets (**Figure 5F**). The significant KEGG pathways enriched in T-cell subsets provided more useful information, which were consistent with the existing knowledge on teleost fish T cells (28, 40) regarding CD4^+^CD8^−^ and CD4^−^CD8^+^ T cells playing different roles in cell-mediated immunity.

In summary, the data presented here using 10× Genomics scRNA-seq provided a rich resource to define the leukocyte subsets in teleost fish AK. The specific gene expression profiles in the healthy fish leukocyte subsets serve as a foundation for studying cell differentiation under stimulation by pathogens or other stimuli. Our study provided potential markers for identifying the myeloid and lymphoid cell types. The highlighted diversity of the B- and T-cell subsets may provide further insights for exploring the maturation and differentiation of B and T cells in teleost fish.

## Data Availability Statement

The sequencing data is available at the NCBI with BioProject accession number PRJNA766837.

## Ethics Statement

The animal study was reviewed and approved by the University Animal Care and Use Committee of the South China Normal University.

## Author Contributions

JY and LW designed the research and provided funding support. LW investigated, collected, and analyzed the data and wrote the original manuscript. AG, LL, and JC fed the animals, helped perform the sample preparation, and reviewed the manuscript. JL contributed to suggestion and critical reading of the manuscript. JY and LW revised and edited the final manuscript. All authors read and approved the submitted version.

## Funding

This study was supported by the National Natural Science Foundation of China (32102827 and 31972818), the Natural Science Foundation of Guangdong Province, China (2019A1515012065), China Postdoctoral Science Foundation (2019M662959), and Guangdong Basic and Applied Basic Research Foundation (2019A1515110987). Special fund for promoting economic development (for modern fishery development) of Guangdong Province (grant number 2019A4).

## Conflict of Interest

The authors declare that the research was conducted in the absence of any commercial or financial relationships that could be construed as a potential conflict of interest.

## Publisher’s Note

All claims expressed in this article are solely those of the authors and do not necessarily represent those of their affiliated organizations, or those of the publisher, the editors and the reviewers. Any product that may be evaluated in this article, or claim that may be made by its manufacturer, is not guaranteed or endorsed by the publisher.
