# Supplementary material for: A Single-Cell Transcriptome Profiling of Anterior Kidney Leukocytes From Nile Tilapia (Oreochromis niloticus)
Source: Front Immunol. 2021 Dec 28;12:783196. doi: 10.3389/fimmu.2021.783196 (PMC8750066; doi:10.3389/fimmu.2021.783196)

Supplementary Figures

Supplementary Figure 1 Single cell sequencing quality control. (A) Identification of the effective cell number of the sample. The X_axis is the number of barcodes, and the Y_axis is the UMI counts. Green line is the effective cells corresponding to barcodes, and the gray line is the background noise. (B) The basic information of sample cell before and after filtering, including the total number of genes (nFeature_RNA), the total number of UMIs (nCount_RNA) and the percentage of reads mapping mitochondrial genes (Percent.mito). (C) The scatter plot of sample cell basic information before and after filtering, including the relationship between nCount_RNA and nFeature_RNA, and between nCount_RNA and percent_mito. The numbers above the graph are Pearson correlation coefficients.

Supplementary Figure 2 The clusters of identified granulocytes and the expression patterns of putative marker genes. (A) A total of 8 clusters were identified in granulocytes and shown with UMAP space. The clusters were obtained with resolution = 0.5. (B) The top five differently expressed genes for each cluster were shown in heatmap. (C) The putative gene markers for granulocyte subsets were used for identifying the obtained 8 clusters in bubble chart. (D) Further putative gene markers were used for identifying different types of granulocytes in clusters were shown in bubble chart.

Supplementary Figure 3 The clusters of identified macrophages and the expression patterns of putative marker genes. (A) A total of 4 clusters were identified in macrophages and shown with UMAP space. The clusters were obtained with resolution = 0.5. (B) The top five differently expressed genes for each cluster were shown in heatmap. (C) The putative gene markers for M1 and M2 populations identification were shown in bubble chart. (D) More putative gene markers for different types of macrophages in clusters were shown in bubble chart.

Supplementary Figure 4 The clusters of identified dendritic cells (DCs), the expression patterns of putative marker genes and cluster 1 Gene Ontology (GO) biological process (BP) enrichment. (A) A total of 3 clusters were identified in DCs and shown in UMAP space. The clusters were obtained with resolution = 0.5. (B) The top five differently expressed genes for

each cluster were shown in heatmap. (C) The putative gene markers of different types of DCs in clusters were shown in bubble chart. (D) The top three terms of GO-BP in cluster 1.

Supplementary Figure 5 Kyoto Encyclopedia of Genes and Genomes (KEGG) pathways enrichment in B cell subsets. (A) The top 10 most significant KEGG pathways for each B cell subset. (B) Bubble chart of the related genes in enriched pathway of antigen processing and presentation in B cell subsets.

Supplementary Figure 6 Heatmap of TFs gene expression analysis in B cell development.

Supplementary Figure 7 The top 10 most significant Gene Ontology (GO) terms within biological process (BP) category for each T cell subset.

Supplementary Figure 8 Heatmap of the putative marker genes for $CD4^{+}$ $CD8^{-}$ (T-helper, Th) and $CD4^{-}CD8^{+}$ (Cytotoxic T, Tc) T cell subsets. (A) $CD4^{+}CD8^{-}$ T cells were classified into 4 clusters with resolution = 0.5. Genes for the identification of Th1, Th2, Th6, Th17, Th22 and regulatory T cell (Treg) were shown in heatmap. (B) $CD4^{-}CD8^{+}$ T cells were classified into 2 clusters with resolution = 0.5. Genes for the identification of Tc1, Tc2, Tc9 and Tc17 were shown with heatmap.

Supplementary Figure 9 Heatmap of TFs gene expression analysis in T cell development.

Supplementary Figure 1

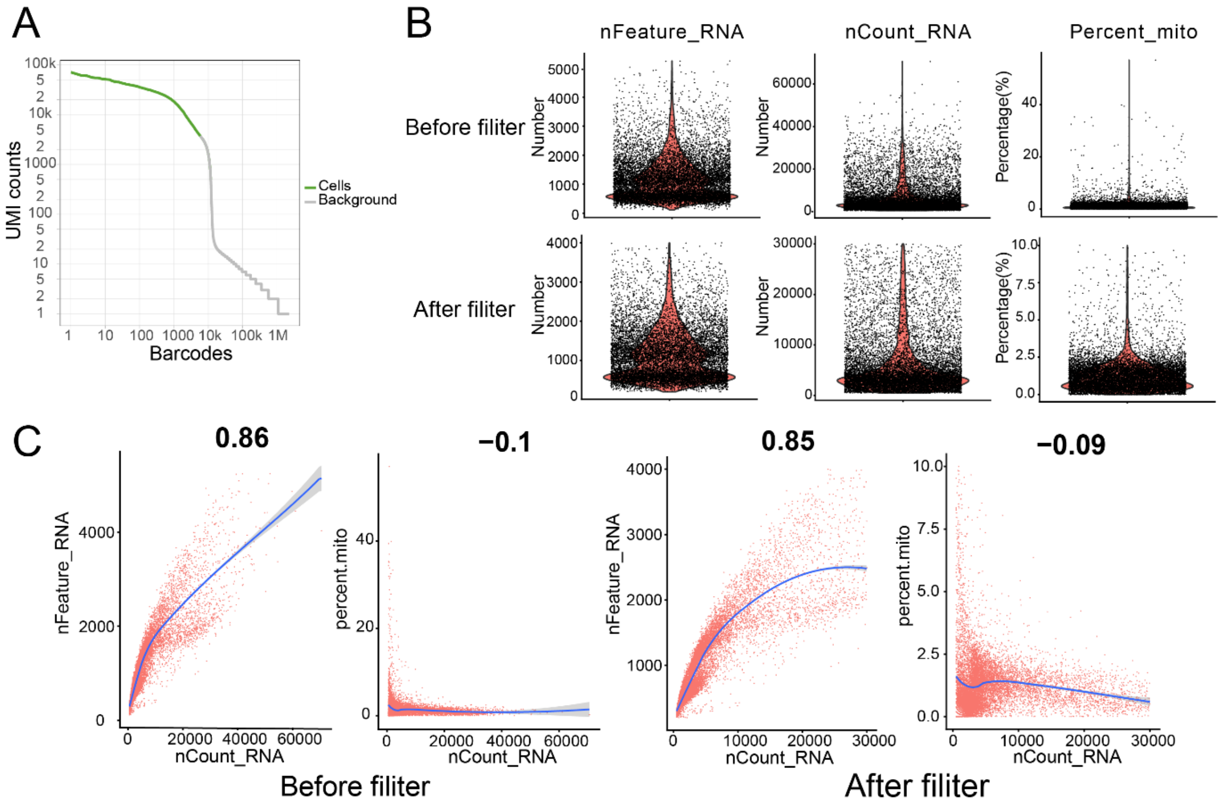

Supplementary Figure 2

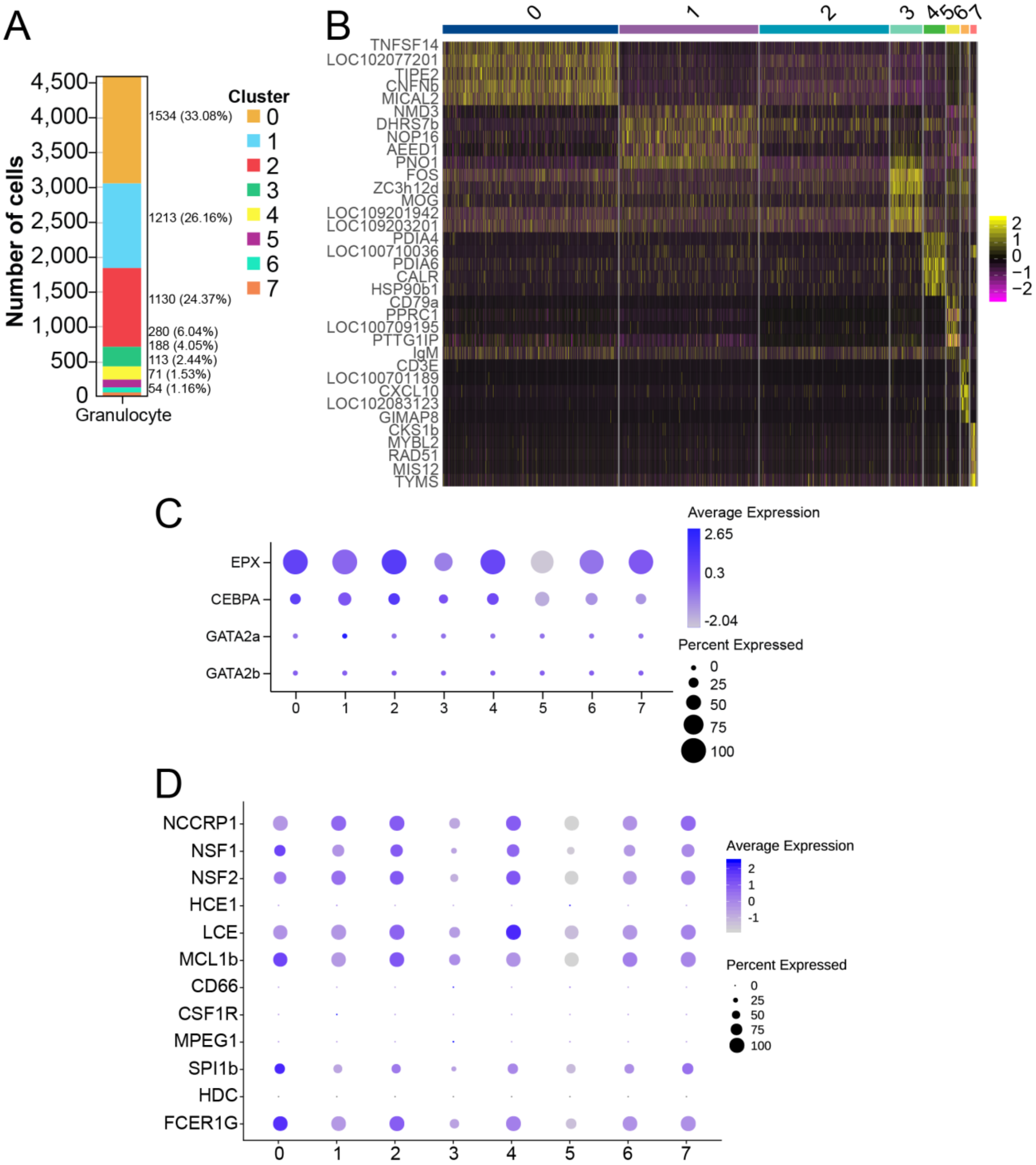

Supplementary Figure 3

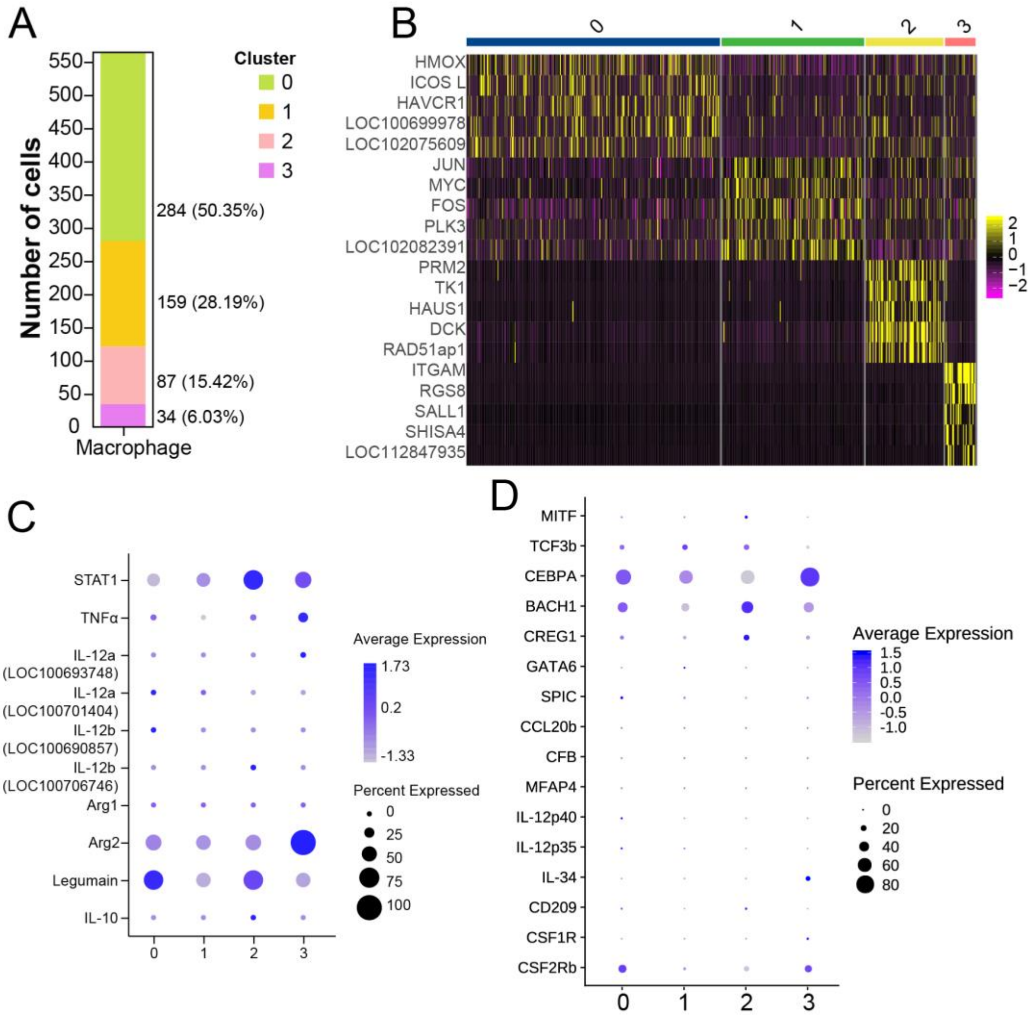

Supplementary Figure 4

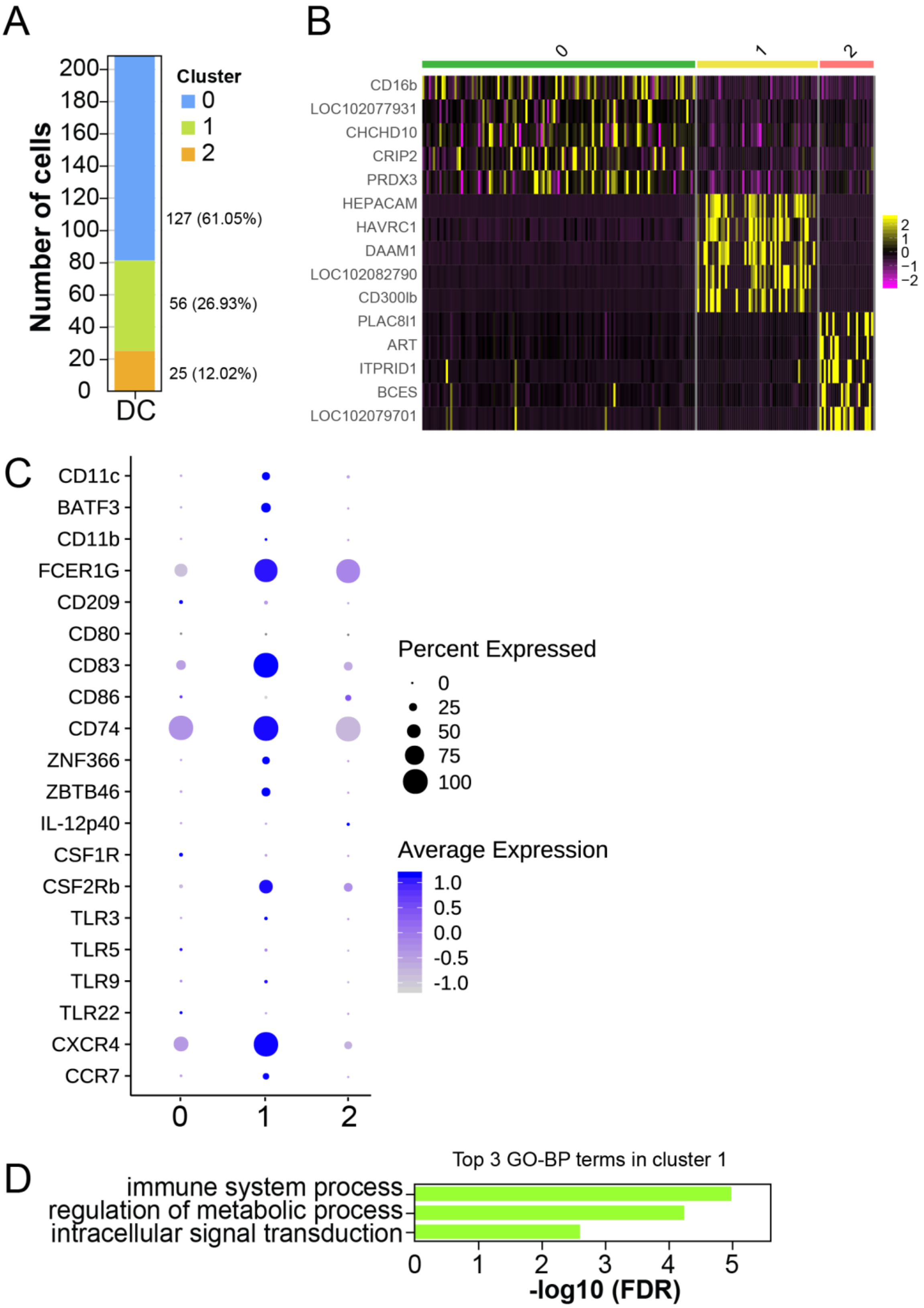

Supplementary Figure 5

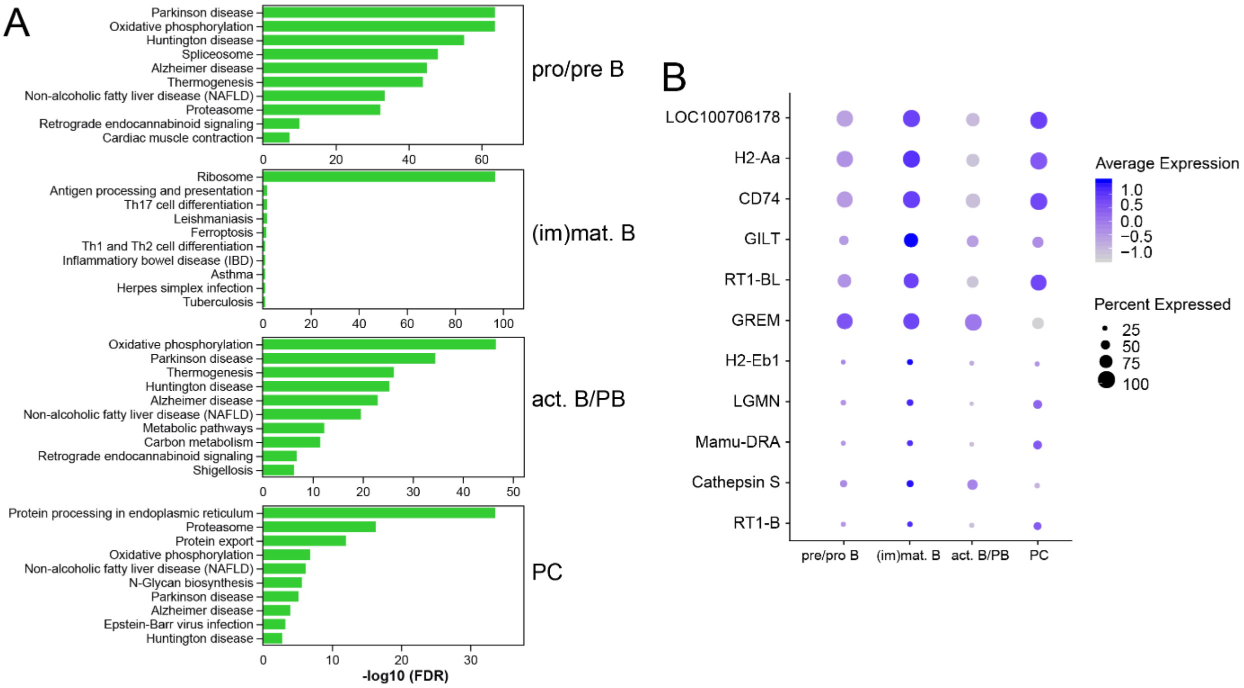

Supplementary Figure 6

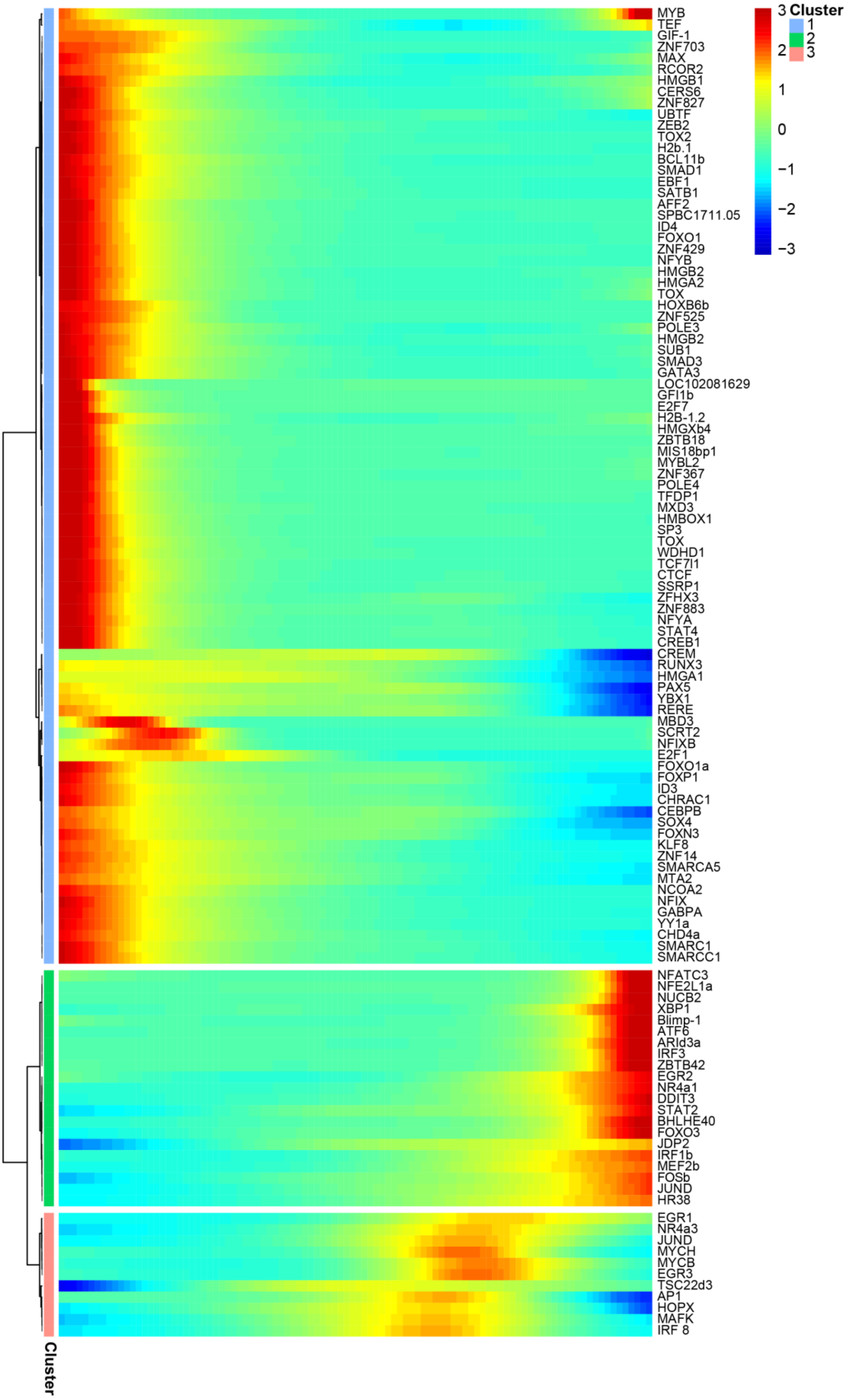

Supplementary Figure 7

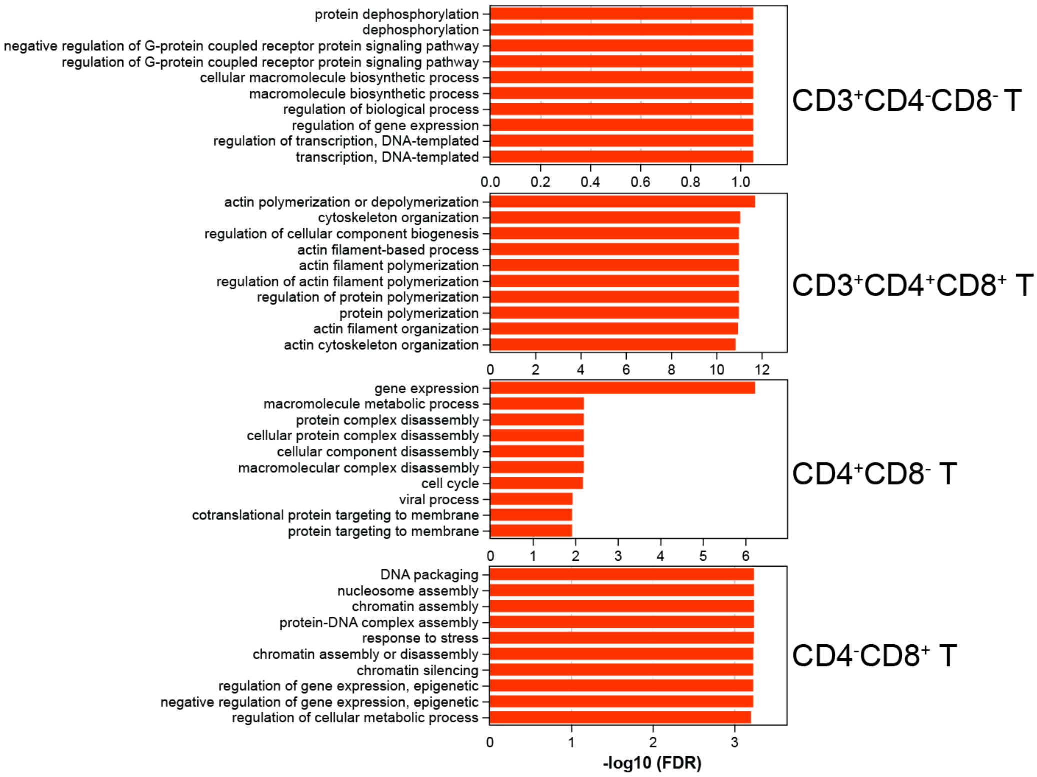

Supplementary Figure 8

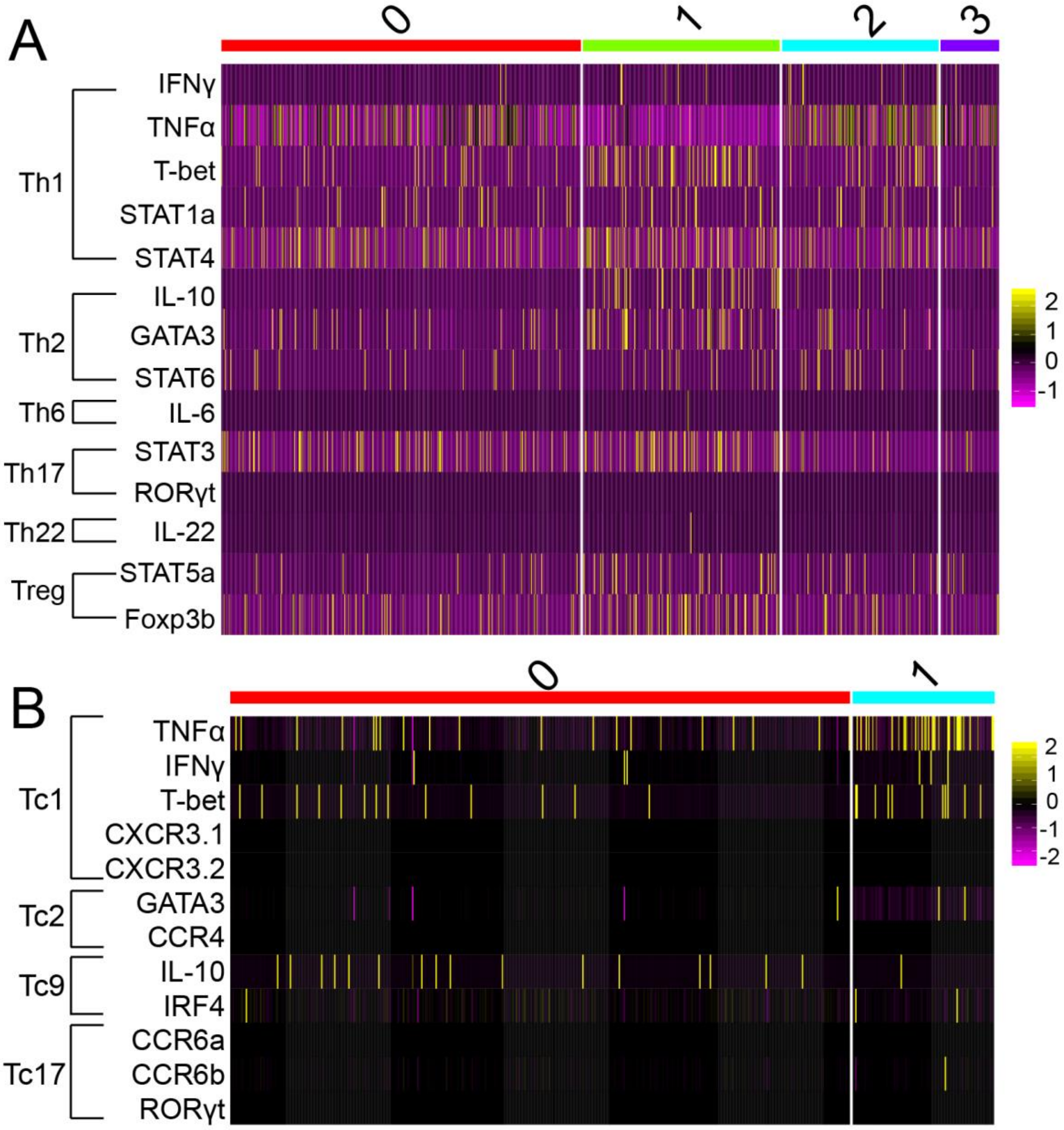

Supplementary Figure 9

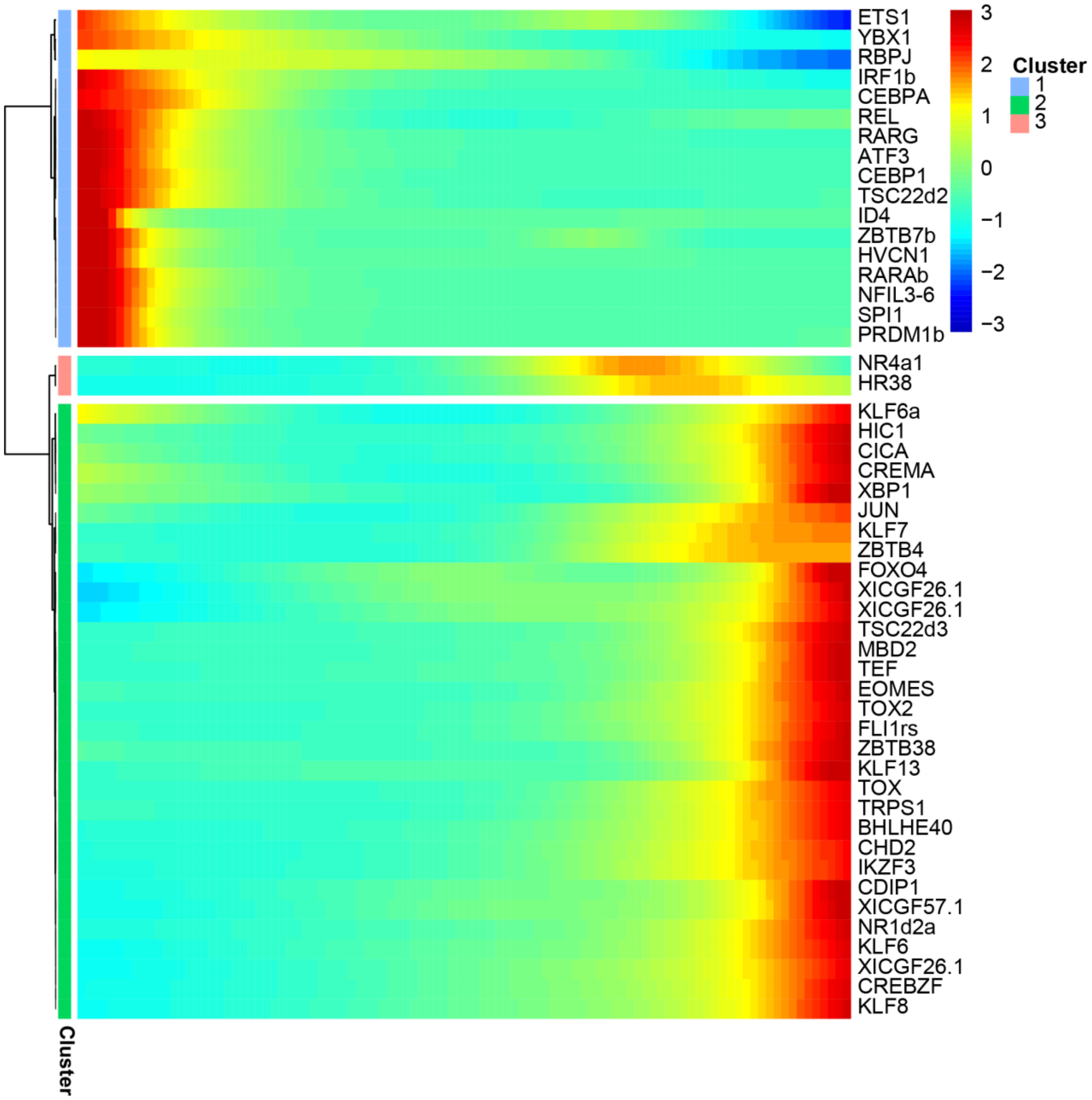

Supplement: Supplementary file 1 [file Presentation_1.pdf]
